# Revisiting Surgical Strategies for Hepatocellular Carcinoma With Microvascular Invasion

**DOI:** 10.3389/fonc.2021.691354

**Published:** 2021-05-27

**Authors:** Er-lei Zhang, Qi Cheng, Zhi-yong Huang, Wei Dong

**Affiliations:** ^1^ Hepatic Surgery Center, Tongji Hospital, Tongji Medical College Huazhong University of Science and Technology, Wuhan, China; ^2^ Key Laboratory of Organ Transplantation, Chinese Academy of Medical Sciences, Wuhan, China

**Keywords:** microvascular invasion, hepatocellular carcinoma, liver resection, liver transplantation, surgical strategy, sub-classification

## Abstract

Although liver resection (LR) and liver transplantation (LT) are widely considered as potentially curative therapies for selected patients with hepatocellular carcinoma (HCC); however, there is still high risk of tumor recurrence in majority of HCC patients. Previous studies demonstrated that the presence of microvascular invasion (MVI), which was defined as the presence of tumor emboli within the vessels adjacent to HCC, was one of the key factors of early HCC recurrence and poor surgical outcomes after LR or LT. In this review, we evaluated the impact of current MVI status on surgical outcomes after curative therapies and aimed to explore the surgical strategies for HCC based on different MVI status with evidence from pathological examination. Surgical outcomes of HCC patients with MVI have been described as a varied range after curative therapies due to a broad spectrum of current definitions for MVI. Therefore, an international consensus on the validated definition of MVI in HCC is urgently needed to provide a more consistent evaluation and reliable prediction of surgical outcomes for HCC patients after curative treatments. We concluded that MVI should be further sub-classified into MI (microvessel invasion) and MPVI (microscopic portal vein invasion); for HCC patients with MPVI, local R0 resection with a narrow or wide surgical margin will get the same surgical results. However, for HCC patients with MI, local surgical resection with a wide and negative surgical margin will get better surgical outcomes. Nowadays, MVI status can only be reliably confirmed by histopathologic evaluation of surgical specimens, limiting its clinical application. Taken together, preoperative assessment of MVI is of utmost significance for selecting a reasonable surgical modality and greatly improving the surgical outcomes of HCC patients, especially in those with liver cirrhosis.

## Introduction

Hepatocellular carcinoma (HCC) is one of the most common malignancies and ranks the third most frequent causes of cancer-related death worldwide (1). China alone accounts for more than half of the new cases in the world (2). Liver resection (LR) and liver transplantation (LT) remain as the first-choice treatments for patients with early-stage HCC. Unfortunately, more than 50% of HCC patients experience recurrence within 5 years after curative treatments, contributing to an increase in the number of tumor-related deaths (3–5). In previous studies, microscopic vascular invasion (MVI), which was always defined as tumor cells invaded to the microscopic vessels in the surrounding liver tissues contiguous to the tumor, had been demonstrated as a strong risk factor associated with tumor recurrence and poor overall survival (OS) among HCC patients after LR or LT (6–15). In addition, MVI was considered as one of the most important prognostic factors within the T criteria in the 8th edition of the American Joint Committee on Cancer (AJCC) staging system (16). However, because of the wide spectrum of current definitions of MVI, other studies indicated that MVI did not affect the long-term surgical outcomes after curative therapies for HCC patients, especially for those with early-stage HCC (17–19). The effect of MVI on long-term surgical outcomes is still in debate. In order to improve surgical outcomes, anatomic resection (AR, complete removal of tumor-bearing portal territory) and wide surgical margin were pursued by liver surgeons, which could theoretically improve the “radical cure” of tumors and decrease the tumor recurrence rates. However, the comparison of clinical efficacy between AR and non-anatomic resection (NAR) had been discussed in the past few decades, making it more clear of their indication based on tumor biological features and underlying liver cirrhosis, but yet needed to be completed. For HCC patients with MVI, how to select these surgical techniques remains unclear. Some studies stressed the importance of the surgical margin width, arguing that AR was not necessary when a wide (≥1 cm) surgical margin can be attained (20, 21). For HCC patients with MVI, wide surgical margin will improve long-term surgical outcomes after LR (9, 22). A recent study indicated that AR with a negative 0-cm surgical margin was not associated with poor OS compared with a negative and wide surgical margin. On the contrary, NAR with a negative 0-cm margin was associated with worse surgical outcomes, suggesting that wide surgical margin should be necessary in HCC patients receiving NAR (23). Up to now, MVI can only be precisely evaluated by histopathologic examination of surgical specimens which limited its clinical application. However, with increasing recognition of MVI and its prognostic value on surgical outcomes of HCC, preoperative MVI prediction systems for surgical decision making have become a hot topic in recent researches. In recent years, some studies have reported the possibility of imaging findings to predict the MVI of HCC (24–27). However, it still has a limited capacity to detect MVI and the precise detection of MVI needs further histological identification (28). This review aims to investigate the impact of current MVI status on surgical outcomes after curative therapy and sheds new light on the surgical strategies for HCC based on different MVI status with evidence from pathological examination.

## The Definition and Sub-Classification of MVI

MVI is a histological feature, and it is acknowledged as tumor cells invading into a portal vein, hepatic vein, or a large capsular vessel of the surrounding hepatic tissues, partially or totally lined by endothelial cells visible only by microscopy (11). Previous studies indicated that the incidence of MVI in HCC patients ranged from 15% to 74.4% (6, 29, 30), and its incidence was positively associated with increasing size of HCC, suggesting that tumor size was an important predictive factor for MVI (31). This wide difference is partly explained by the selection bias of included patients and varied definition of MVI in HCC. MVI includes a wide spectrum, ranging from invasion of a single small vessel around the tumor capsule to micro-portal vein invasion (6). With increasing recognition of MVI and its prognostic value on surgical outcomes, preoperative MVI prediction models for surgical decision making in HCC have become a hot topic in recent years (10, 11, 32). However, due to the broad range of current definitions for MVI, one would also expect a varied range of surgical outcomes in HCC patients with MVI undergoing LR or LT (7, 31, 33, 34).

In the past few years, several studies had attempted to put forward MVI classifications according to the number of invaded vessels, number of tumor cells, distance of invaded vessel to tumor edge and subtypes of invaded vessels (10, 35–40). In the practical guidelines for the pathological diagnosis of HCC developed by China (35), the presence of MVI was recommended to be evaluated in all tissue sections and graded according to the risk stratification based on the number and distribution as follows: no MVI; low-risk (M1): <5 MVI and ≤1 cm away from the tumor tissues; and high-risk (M2): >5 MVI or >1 cm away from the tumor tissues. Roayaie et al. (10) proposed a novel classification of MVI in HCC patients, which included invasion of a vessel with a muscular wall and a vessel invasion that was more than 1 cm away from the tumor tissues. Sumie et al. (39) sub-classified MVI into three grades according to the number of vessels invaded: no vascular invasion (NVI), mild MVI (1–5 vessels), and severe MVI (>5 vessels). Another study from China sub-classified MVI into non-MVI, low-MVI (the number of invaded vessels ≤5, the number of invaded carcinoma cells ≤50 and the distance of invasion from tumor edge ≤1 cm) and high-MVI (the number of invaded vessels >5, the number of invaded carcinoma cells >50 and the distance of invasion from tumor edge >1 cm) (38). This classification was similar with a study from Japan (40). Nonetheless, the definitions of MVI in these sub-classification systems were obscure. They defined MVI as follows: clusters of tumor cells were observed in the portal vein accompanied with hepatic artery and bile duct in the portal tract, and clusters of tumor cells in the vessels in fibrous capsule of HCC were also defined as MVI (40). In addition, these classification systems were relatively complicated to generalize in real pathologic circumstances and had not been revalidated in other research. A summary of the current MVI classification had been summarized by Erstad and colleagues (41). A recent study by Kang et al. (7) suggested that MVI in excised specimens should be further sub-classified into microvessel (which was defined as newly developed microvascular structures in the tumor capsule or fibrotic peritumoral non-tumor liver, these microvessels were not portal veins, hepatic veins or hepatic arteries) invasion (MI) and microscopic portal vein invasion (MPVI). Their results indicated that MPVI was associated with more aggressive clinic-pathologic features and poorer surgical outcomes compared with HCC patients with MI. Therefore, they recommended that the original MVI classification should be divided into MI and MPVI and it needed further research to validate their findings. However, the number of surgical specimen slides of examined and surgical types also affect the assessment of MVI and inadequate specimen may induce a false-negative evaluation (40, 42). In this regard, it is urgent to establish a universal criterion for the pathological and clinical study of MVI.

## MVI and Surgical Outcomes

### Liver Resection (LR)

MVI is widely acknowledged as an expression of aggressive biological behavior of HCC, and is currently one of the most critical factors associated with adverse prognosis after curative resection. The 5-year Disease Free Survival (DFS) rate ranges from 7.5% to 48% and the corresponding 5-year OS rate is 38.4% to 66% in HCC patients with MVI (7, 9, 14, 22, 31, 33, 34, 43–47). The reasons for this broad range may be due to a lack of consensus on the definition of MVI and selected patients with different tumor size in the above studies. Although there is an agreement in the definition of MVI on some histologic features, such as the presence of tumor cells in portal vessels, large vessels of the fibrotic capsule, there are still some controversies in other aspects, such as the distance from the invaded vessels to the edge of tumor, the number of invaded vessels (6). It remains unclear which biological features are the most important factors for surgical prognosis. In this review, we allow the different definitions of MVI and aim to evaluate the impact of MVI status on surgical outcomes. HCC patients with MVI have been demonstrated to have a wide range of surgical outcomes. A meta-analysis analyzed 1501 HCC patients undergoing LR, and addressed the prognostic impact of MVI on surgical outcomes, the results indicated that the presence of MVI reduced their 5­year DFS rates (RR = 1.51 [1.29–1.77]) (6). A study reported that stage II HCC patients (based on TNM stage) with MVI had similar surgical outcomes compared with stage III HCC patients without MVI, which demonstrated that MVI was a more important factor which affected tumor recurrence and long-term survival (median, OS 4.3 vs 3.9 years, *p* = 0.622) (48). Other studies indicated that HCC patients with MVI had lower RFS and OS rates than those without MVI even for patients with solitary HCC ≤ 2 cm (49, 50). Sumie et al. (39) evaluated whether the classification of MVI based on number of invaded vessels affected tumor recurrence and survival after surgical resection for HCC patients. MVI were stratified into no MVI, mild MVI and severe MVI groups and the DFS rates at 2 years were 75.9%, 47.2%, and 32.7%, respectively (*p*<0.05). The corresponding OS rates at 5 years were 91.5%, 70.4%, and 51.4%, respectively (*p* < 0.05) (39). However, the classification was difficult to apply in clinical practice due to complex histological examination. A recent study sub-classified HCC patients with MVI into MI and MPVI group. Their results indicated that both MI and MPVI were independent prognostic factors for DFS and OS after LR. The 5-year DFS rates were 75%, 45%, and 25% in the NVI, MI, and MPVI groups, respectively (*p*<0.001), whereas the corresponding 5-year OS rates were 90%, 78%, and 55%, respectively (*p* < 0.001) in HCC patients undergoing LR (7). In HCC patients, the spread of tumor cells *via* a portal vein has generally been accepted as the main mechanism for intrahepatic metastasis (10, 51). This mechanism indicated the progress of tumor invasion from MI to MPVI. Accordingly, MPVI showed a higher frequency of early recurrence within 1 year and extrahepatic recurrence than MI (7). Unfortunately, in this study, the authors did not analyze the impact of AR and surgical margin status on the surgical outcomes with MI or MPVI. Studies that assessed the prognostic significance of MVI for long-term outcomes after LR are summarized in **Table 1**.

**Table 1:** MVI prognostic capability in HCC after liver resection.

Author	Vascular type	MVI status	Number	5-year OS (%)	*p*-value	5-year DFS (%)	*p*-value	Published year
Kang et al. (7)	MI+MPVI	NVI	212	90	0.001	75	<0.001	2020
		MI	64	78		45		
		MPVI	36	55		25		
Song et al. (43)	MI+MPVI+MHVI	NVI	206	NA	NA	49.3	<0.001	2019
		MVI	94	NA		28.2		
Wang et al. (34)	MI+MPVI+MHVI	NVI	34	78.3	<0.001	45.7	<0.001	2019
		MVI	30	48.9		7.5		
Nitta et al. (33)	MI+MHVI	NVI	148	66.1	<0.001	28.6	<0.001	2019
		MVI	117	38.4		15.8		
Han et al. (9)	NA	NVI-N*	145	64	<0.05	41	<0.05	2018
		MVI-N*	158	55		30		
Han et al. (9)	NA	NVI-W^#^	300	78	<0.01	58	<0.01	2018
		MVI-W^#^	192	66		48		
Hwang et al. (31)	NA	NVI	1720	80.9	<0.001	47.1	<0.001	2015
		MVI	236	61.2		30.9		
Banerjee et al. (44)	NA	NVI	111	78	<0.001	61	<0.001	2015
		MVI	44	55		33		
Yamashita et al. (18)	MPVI+MHVI+MBDI	NVI	106	87	0.26	72	<0.001	2012
		MVI	43	67		44		
Lim et al. (14)	NA	NVI	197	61.3	<0.001	NA	NA	2011
		MVI	44	36.6		NA		

NA, not available; MI, microvessel invasion; MHVI, microscopic hepatic vein invasion; MPVI, microscopic portal vein invasion; MBDI, microscopic bile duct invasion; N*, narrow surgical margin <1 cm; W^#^, wide surgical margin ≥1 cm.

### Liver Transplantation

Liver transplantation (LT) is generally accepted as the first-line treatment for selected HCC patients for the reason of removing the entire tumor and underlying liver disease. Especially, the severity of cirrhosis at risk for the development of *de novo* HCC is removed (52). Waitlist priority for a LT in America and Europe follows the “sickest-first” principle: decompensated cirrhosis and HCC (53). In order to limit LT to those with poor post-LT outcomes, HCC waitlist priority has mainly relied on two criteria: Milan criteria (solitary tumor <5 cm or up to three nodules and each <3 cm in size, without major vascular invasion) and University of California-San Francisco (UCSF, solitary tumor of 6.5 cm, or three nodules with the largest diameter of 4.5 cm and a total tumor diameter of 8 cm) criteria, to exclude those with high risks of tumor recurrence (54, 55). Studies indicated that HCC patients within the Milan and UCSF criteria had equivalent survival rates and recurrence rates, and surgical outcomes were comparable for MVI-negative patients within or beyond Milan criteria (3.3% versus 4.7%) (13, 56). Accordingly, based on these surveys, LT for HCCs beyond the Milan criteria was an acceptable and life-saving method. Since the high cost, long waiting times on the waiting list of LT, coupled with the allograft shortage, it is of great importance to allocate donor livers to HCC patients with the best opportunity for long-term surgical outcomes.

It is widely accepted that MVI is one of the most important risk factors for tumor recurrence and poor OS among HCC patients within transplantation criteria after LT (7, 33, 36, 57–64). By defining MVI as tumor cells were present within the lumens of veins microscopically, Nitta et al. (33) recently compared the surgical outcomes after LT for MVI-positive HCC patients (n = 134) and MVI-negative HCC patients (n = 238). The 5-year OS rates were 60.9% and 89.9%, respectively (*p*<0.001), the corresponding 5-year DFS rates were 51.4% and 80.6%, respectively (*p* < 0.001). A meta-analysis of 2003 HCC patients who underwent LT indicated that MVI was related to 2.3-fold decrease of 5-year OS rate compared with those without MVI (6). Therefore, MVI was the most common factor used in risk stratification and surveillance for HCC recurrence after LT (65). Bhatti et al. (66) indicated that the estimated 4-year RFS rate in patients with combined AFP > 600 ng/ml and MVI was 0% after living donor LT, while 83% in patients with combined AFP ≤ 600 ng/ml and NVI (*p* < 0.001). Taken together, these findings indicated that MVI should be considered as a surgical contraindication to LT as it represented a waste of allografts. Conversely, a study from America evaluated the impact of MVI on prognosis of patients with HCC ≤ 2 cm after LT; their results indicated that HCC ≤ 2 cm had an excellent prognosis after LT and was not affected by the presence of MVI (67). Chan et al. (58) investigated the survival benefit of primary LT for HCC with MVI and within the up-to-7 criteria (7 as the sum of largest tumor diameter and tumor number). The 5-year OS rate was 85.7%, and was not affected by the presence or absence of MVI (88.2% vs. 85.1%). Therefore, the authors recommended that the presence of MVI should not be a contraindication to LT for HCC patients. It is worth noting that the definition of MVI was not recorded and small number of patients (n=23 and n=17) were MVI-positive, which may be the reason for this discrepancy with the previous studies.

In this regard, more accurate and simple definition of MVI will be needed for assessing the value of LT. Kang et al. (7) investigated whether classification of MVI affected the surgical outcomes after LT in HCC patients. The 5-year DFS rates were 89%, 67.9% and 0% in the NVI, MI and MPVI groups, respectively (*p*<0.001), whereas the 5-year OS rates were 79.1%, 55.0%, and 15.4%, respectively (*p*<0.001). Accurate prediction of MVI before surgery can help liver surgeons to select suitable candidates for LT. When MPVI is predicted, the authors recommended that LT should not be pursued based on the poor surgical outcomes as determined by the classification system proposed (7). A recent study by Carr et al. (68) indicated that the mean overall survival of HCC patients with MPVI was significantly worse than those without MPVI (86.6 versus 110.5 months, *p*=0.007). They further suggested that MPVI was associated with multiple tumor nodules, larger tumor size and higher serum levels of both AFP and gamma-glutamyl transpeptidase (GGT). Although LT for MVI-positive HCCs provides relative good prognosis, nevertheless, it’s associated with poor surgical outcomes compared with other indications for LT. However, LT is still considered as first-line treatment for MVI-positive HCC patients due to the lack of accurate preoperative methods for the prediction of MVI. HCC patients with high-risk MVI waiting for LT may get benefit from preoperative neo-adjuvant treatment. A recent study demonstrated that bridging loco-regional therapy with Y-90 trans-arterial radio-embolization could reduce the incidence of MVI after LT and may improve tumor control and reduce post-LT recurrence (69). Nevertheless, it needs to be further verified in randomized controlled trials.

It is of great importance to support post-LT surveillance for HCC recurrence, because early diagnosis and timely intervention may improve OS for HCC patients with MVI. Accordingly, liver surgeons attempted to find pre-operative biomarkers of MVI that predict recurrence and survival. Liver biopsy using fine-needle aspiration (FNA) could be one method to evaluate the MVI status of HCC patients on the waiting list. However, preoperative detection of MVI *via* biopsy was inaccurate due to intratumoral heterogeneity and sampling error (41, 70), and may increase the risk of tumor recurrence (64). Accordingly, there is an urgent need for a reliable non-invasive method to preoperatively detect the MVI status in the future. Studies which assessed the prognostic significance of MVI for long-term outcomes after LT are summarized in **Table 2**. It should be noted that the definition of MVI was not elaborated in nearly two-thirds of the studies (8/13). Therefore, the criteria for defining MVI are highly variable among these studies, which could affect the final results, thus, a consensus on defining MVI is necessary.

**Table 2:** MVI prognostic capability in HCC after liver transplantation.

Author	Vascular type	MVI status	Number	5-year OS(%)	*p*-value	5-year DFS(%)	*p*-value	Published year
Kang et al. (7)	MI+MPVI	NVI	144	79.1	<0.001	89	<0.001	2020
		MI	40	55		67.9		
		MPVI	13	15.4		0		
Carr et al. (68)	MPVI	NVI	105	74.8	<0.001	NA	NA	2020
		MVI	165	55.3		NA		
Nitta et al. (33)	MI+MPVI+MHVI	NVI	238	89.9	<0.001	80.6	<0.001	2019
		MVI	134	60.9		51.4		
Choi et al. (63)	NA	NVI	184	87.1	<0.05	76.8	<0.05	2017
		MVI	24	64.5		63.3		
Mohamed et al. (67)	NA	NVI	199	94	0.44	NA	NA	2017
		MVI	23	86		NA		
Grąt et al. (62)	NA	NVI	143	NA	NA	85.9	0.001	2017
		MVI	57	NA		55.3		
Donat et al. (64)	NA	NVI	110	69.9	0.007	59.5	0.003	2016
		MVI	41	48.4		32		
Vilchez et al. (61)	MPVI+MHVI	NVI	73	68	0.001	NA	NA	2016
		MVI	22	52		NA		
Agopian et al. (60)	NA	NVI	646	NA	NA	64	<0.001	2015
		MVI	163	NA		44		
Iguchi et al. (36)	MPVI+MHVI	NVI	87	NA	NA	87.5	<0.001	2015
		MVI	38	NA		42.6		
Moon et al. (59)	NA	NVI	112	90.5	<0.001	95.3	<0.001	2012
		MVI	74	58.2		57.2		
Chan et al. (58)	NA	NVI	60	85.1	NS	86.4	NS	2012
		MVI	17	88.2		88.2		
McHugh et al. (57)	NA	NVI	71	72	0.001	NA	NA	2010
		MVI	30	40		NA		

NA, not available; NS, no significance.

## MVI and Selection of Surgical Type

### AR or NAR

Over the past few decades, debate about the superiority of AR compared with NAR for providing better surgical prognosis had never stopped. Previous studies demonstrated that AR obtained better OS and recurrence free survival (RFS) compared with non-anatomical resection (NAR) for HCC patients after LR (15, 71–77). Two recent meta-analyses demonstrated that AR seemed to offer better surgical outcomes versus NAR among HCC patients undergoing LR, especially for HCC patients without cirrhosis and small solitary tumors, although AR had similar perioperative morbidity and mortality versus NAR (78, 79). However, other studies showed different results (80–84). A nationwide survey from Japan indicated that there was no significant difference in surgical outcomes after LR for solitary HCC between the AR and NAR groups, but the recurrence rates in the AR group were significantly better in HCC patients with a tumor diameter of 2 to 5 cm compared with those in the NAR group (85). Since HCC tumor cells are prone to spread through the portal venous system, theoretically, the AR method may prevent tumor cells spreading *via* portal vein invasion in HCC (75). Nevertheless, the effects and benefits of AR for HCC with MVI remain controversial (15, 76, 82). For HCC patients with MVI which was defined as the presence of tumor cells in a portal vein, hepatic vein, or capsular vessel of the surrounding liver tissue lined by endothelium that was only visible microscopically, propensity score matching analysis was used to eliminate selection bias. AR significantly suppressed tumor recurrence after LR, whereas the OS rates were similar between the two groups (15, 22, 86). Nonetheless, other studies failed to demonstrate these benefits when performing AR (8, 18, 82). The conflicting results were easy to understand because the OS of HCC patients was also affected by the tumor characteristics, hepatitis virus infection status and liver cirrhosis except surgical factors (87). In addition, it was noteworthy that the definitions of MVI were different, which may be responsible for the inconsistent conclusions. Yamashita et al. (18) evaluated the prognoses of HCC patients accompanied with micro-invasion (such as portal venous, hepatic vein or bile duct infiltration microscopically); the results indicated that there was no survival benefit of AR compared with NAR for patients with HCC ≤ 2 cm. Micro-metastases could spread *via* invasion of portal venous system and then develop to form microsatellite nodules even at an early stage (9). This suggested that AR was preferred for HCC patients with solitary tumor while NAR with adequate surgical margin could be used as an alternative treatment in HCC patients with limited liver function (51). Previous study showed that majority of classical HCCs have tumor capsules, making it seems impossible to be a microscopically positive surgical margin, although the surgical margin width was 0 cm when performing AR. However, NAR with a wide surgical margin will be needed for HCC patients aiming to preserve functional liver parenchyma as much as possible, especially in patients with liver cirrhosis (23). Unfortunately, this study did not consider the MVI status. For HCC patients with MVI, AR combined with a surgical margin ≥1 cm provided better surgical outcomes compared with NAR with surgical margin less than 1 cm (*p*=0.001), whereas AR was not necessary for HCC patients without MVI (47, 88). A multi-institutional study from Japan evaluated the value of AR for HCC with MPVI, the results demonstrated that there were no significant differences in long-term surgical outcomes between the AR and NAR groups using a propensity score-matched analysis (8). Especially, for tumors about 2 to 5 cm in size, AR still failed to gain a better OS or RFS than NAR, although the NAR group had higher proportions of hepatitis c virus (HCV), Child-Pugh class B, liver damage class B and worse ICG-R15 results than the AR group. A summary of studies in the past ten years which compared both recurrence and survival of surgical type (AR or NAR) for HCC patients with MVI are displayed in **Table 3**.

**Table 3:** MVI prognostic capability in HCC receiving AR or NAR.

Author	Vascular type	Resection	Number	5-year OS(%)	p-value	5-year DFS(%)	p-value	Published year
Hidaka et al. (8)	MPVI	AR	422	62.3	NS	38.2	NS	2020
		NAR	124	66.7		36.6		
Hidaka et al. (8)	MPVI	AR-PSM	86	64.5	NS	37	NS	2020
		NAR-PSM	86	65.3		42.2		
Shi et al. (22)	MI+MPVI+MHVI	AR	118	NA	NA	45	0.001	2019
		NAR	113	NA		20		
Zhong et al. (86)	MI+MPVI+MHVI	AR	100	51.5	0.301	42	0.039	2019
		NAR	170	42.4		26.4		
Zhao et al. (15)	MI+MPVI+MHVI	AR	45	52	0.277	39	0.016	2017
		NAR	47	42		20		
Matsumoto et al. (76)	MPVI	AR	74	46.1	0.002	33.8	0.001	2016
		NAR	23	16.3		0		
Marubashi et al. (82)	NA	AR	110	NA	NA	50	0.312^#^	2015
		NAR	83	NA		43		
Yamashita et al. (18)	MPVI+MHVI+MBDI	AR	13	88	0.84	47	0.92	2012
		NAR	30	65		23		

NA, not available; NS, no significance; AR, anatomical resection; NAR, non-anatomical resection; PSM, propensity score match.

^#^2-year DFS.

### Wide or Narrow Surgical Margin

Whether surgical resection should be performed with wide or narrow surgical margin remains controversial in MVI-positive HCC patients. Several studies evaluated the relationship among surgical margin width, surgical outcomes and MVI status using measurement cut-offs for surgical margin width of 0.2, 0.5, and 1 cm (9, 18, 46, 47). Since the incidence of MVI was correlated with tumor size, and MVI were reported to mainly present in the surgical margin adjacent to tumor, increased surgical margin width may improve surgical outcomes (31). Yamashita et al. (18) found that the presence of MVI was associated with poor surgical outcome in 5-year DFS rate (44% vs 72%, *p*<0.01) by evaluating 149 patients with surgically excised HCC<2 cm in diameter, however, this negative effect was neutralized with a wider surgical margin (≥0.5 cm). Wang et al. (46) evaluated the influence of surgical margin on the prognosis of patients with HCC ≤ 5 cm and found that both the RFS and OS rates showed no significant difference in narrow (≤0.2 cm) and wide-margin (>0.2 cm) LR for the patients without MVI. However, for HCC patients with MVI, the narrow-margin LR showed poorer surgical outcomes than the wide-margin LR. The 1-, 3-, and 5-year RFS rates in the two groups were 63.3%, 32.8%, and 25.4% versus 74.9%, 60.6%, and 56.7%, respectively (*p* < 0.001). The 1-, 3-, and 5-year OS rates in the two groups were 89.9%, 67.8%, and 56.8% versus 97.1%, 84.2%, and 76.3%, respectively (*p*= 0.001). These studies indicated that a wider surgical margin could eliminate the peripheral intrahepatic micro-metastases, thus prevent early tumor recurrence. A recent study assessed the impact of hepatic surgical margin width on long-term outcomes for solitary HCC patients who underwent a negative margin curative LR. Margin width was categorized as “narrow” (≤ 0.3 cm), “intermediate” (0.3–1.0 cm), or “wide” (> 1.0 cm), and there was no significant survival difference among the three groups regardless of MVI status. It should be noted that only 23.6% of HCC patients accompanied with MVI and nearly 70% of the patients underwent AR in this study, which may contribute to the better surgical outcomes. They recommended that narrow surgical margins seemed to be oncologically safe and the feasibility of achieving wide surgical margins should not be a determinant of resectability (89). The average tumor size was approximately 5.2 cm in this study, indicating that a wider surgical margin did not translate to better surgical outcomes for larger HCC patients with MVI, probably due to a greater burden of micro-metastases. However, another recent study indicated that more than 0.7-cm surgical margin was important to prevent early recurrence among HCC patients with MVI, no tumor capsule and alpha-fetoprotein (AFP) levels ≥100 ng/ml (90). The reason for these inconsistent results may be partly due to different definitions of MVI and varied surgical margins. Actually, more than 80% of HCC patients that undergo LR are associated with liver cirrhosis in China (91, 92). For HCC patients with advanced liver cirrhosis, preserving adequate liver tissue is more important for surgical outcomes and it is less likely to undergo major surgical resection. Furthermore, the correlation between liver cirrhosis and MVI remains unclear. A summary of studies in the past ten years which evaluated the impact of surgical margin status on surgical outcomes for HCC patients with MVI are showed in **Table 4**.

**Table 4:** MVI prognostic capability in HCC with different width of surgical margin.

Author	Vascular type	Surgical margin	Number	5-year OS(%)	*p*-value	5-year DFS(%)	*p*-value	Published year
Wang et al. (46)	MI+MPVI+MHVI	≤0.2 cm	130	56.8	0.01	25.4	<0.01	2020
		>;0.2 cm	132	76.3		60.6		
Han et al. (9)	NA	<1 cm	158	55	<0.01	30	<0.01	2019
		≥1 cm	192	66		48		
Shi et al. (22)	MI+MPVI+MHVI	≥1 cm	105	50	0.01	NA	0.006	2019
		<1 cm	126	25		NA		
Yang et al. (47)	MI+MPVI+MHVI	<1 cm	545	25	<0.01	14.1	<0.01	2019
		≥1 cm	384	44.9		38.9		
Yamashita et al. (18)	MPVI+MHVI+MBDI	≥0.5 cm	20	61	0.95	33	0.04	2012
		<0.5 cm	23	73		21		

NA, not available.

**Figure 1:**
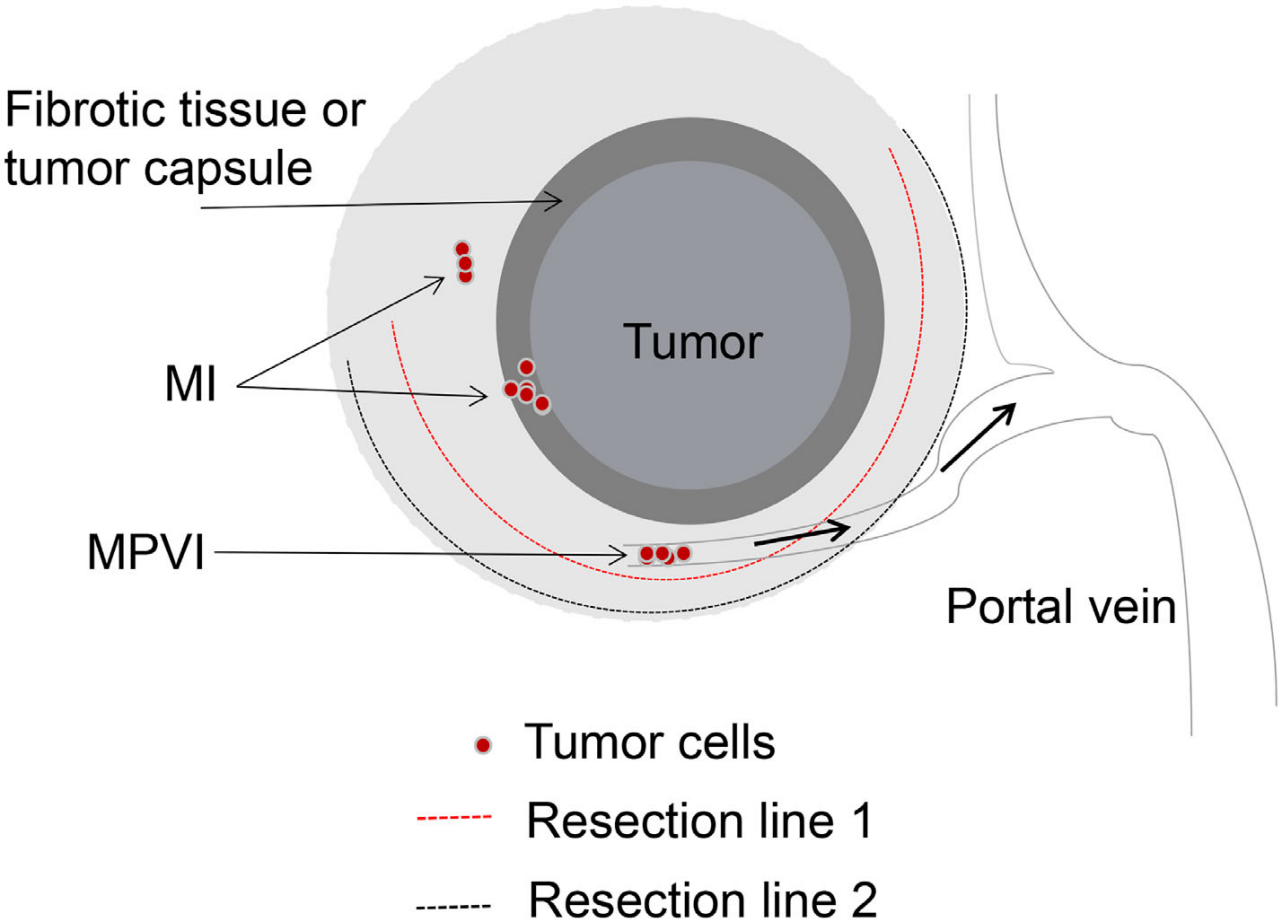
Surgical strategy of LR based on the sub-classification of MVI. MI, Tumor cells in the microvascular space of the tumor capsule or compressed and fibrotic liver tissue, adjacent to the tumor mass. MPVI, Tumor cells in the portal vein, away from the tumor mass. Both of resection line 1 and 2 could completely remove the tumor and tumor vascular invasion for patients with MI, however, it’s unavailable for patients with MPVI.

Some authors emphasized the significance of the surgical margin width, arguing that AR was not necessarily needed when a wide and negative surgical margin can be obtained (20, 21, 90). The impact of surgical margin width on long-term outcomes of HCC patients with MVI underwent LR has not been evaluated in relation to the type of LR performed. Our preliminary results indicated that 2-year recurrence rate in the MPVI group was significantly higher than that in the MI and NVI groups after LR (49.8% vs. 20.3% vs. 5.8%, *p*<0.01). HCC patients in MI group with a wide surgical margin (≥1 cm) showed better surgical outcomes than those with a narrow surgical margin (<1 cm). It was interesting to note that patients with MPVI experienced similar surgical outcomes irrespective of wide or narrow surgical margin. In addition, AR did not show better surgical outcomes after curative LR compared with NAR, which was consistent with previous results (8). Taken together with our experience and the results from previous studies, for HCC patients with MVI, AR or NAR and wide or narrow surgical margin should be performed individually. MVI should be sub-classified into MI and MPVI, for those HCC patients with MPVI, microvascular tumor thrombus at the transection site may disseminate to larger vessels *via* portal venous system and result in intrahepatic recurrence at a distance from the surgical margin in the remnant liver, which should be considered to be a systemic disease. Local resection with a wide or narrow surgical margin will get the same surgical results. However, for those HCC patients with MI which could be considered as a local disease, NAR with a wide and negative surgical margin will acquire relative better surgical outcomes (shown in **Figure 1**).

## Conclusion

The presence of MVI is highly associated with adverse biological tumor behavior in HCC and is a critical determinant of HCC recurrence after LR or LT. Surgical outcomes of HCC patients with MVI have been described as a varied range after curative therapies due to the broad spectrum of current definitions for MVI. An international consensus on the validated definition of MVI in HCC is urgently needed which may provide a more consistent assessment and reliable prediction of surgical outcomes for HCC patients after curative treatments. MVI should be further sub-classified into MI and MPVI, for HCC patients with MPVI, local R0 resection with a narrow or wide surgical margin will get the same surgical results. However, for HCC patients with MI, local surgical resection with a wide and negative surgical margin will acquire better surgical outcomes. Unfortunately, MVI can only be precisely identified according to the postoperative examination of surgical specimens, limiting its potential value in guiding the personalized therapy. In the recent years, with the development of imaging technologies, application of axial imaging using CT, MRI, and PET/CT together with the novel serum biomarkers and molecular characterization of biopsy tissue provided more reliable methods for preoperatively predicting MVI status. Precise preoperative measurement of MVI is urgently needed, as it is helpful to select a reasonable surgical modality and greatly improve the surgical outcomes of HCC patients, especially in those with liver cirrhosis.

## Author Contributions

E-LZ and WD made the concept and design. QC drew the figure. E-LZ drafted the manuscript. Z-YH and WD critically revised the manuscript for the final version. All authors contributed to the article and approved the submitted version.

## Funding

This work was supported by the grants from National Natural Science Foundation of China (81902839) and National Science and Technology Major Project of China (2017ZX10203207-002).

## Conflict of Interest

The authors declare that the research was conducted in the absence of any commercial or financial relationships that could be construed as a potential conflict of interest.
